# Associations of PD-L1 expression status, tumor histology and patient ethnic background with immunotherapy outcomes in patients with advanced esophageal cancer

**DOI:** 10.3389/fphar.2026.1879335

**Published:** 2026-07-17

**Authors:** Xuehua Cai, Junxing Xie, Bin Zhao

**Affiliations:** Quanzhou First Hospital Affiliated to Fujian Medical University, Quanzhou, China

**Keywords:** clinicopathological characteristics, esophageal cancer, immunotherapy, overall survival, PD-L1

## Abstract

**Background:**

Currently, immune checkpoint inhibitors (ICIs) have become a mainstay in treating advanced esophageal cancer (EC), yet the predictive significance of routinely collected clinicopathological characteristics for overall survival (OS) remains uncertain.

**Methods:**

We searched EMBASE, MEDLINE, Cochrane Library, Web of Science, and ClinicalTrials.gov for phase III randomized controlled trials (RCTs). Eligible studies reported OS for the overall population and subgroups stratified by various clinicopathological features. Pooled hazard ratios (HRs) and their 95% confidence intervals (CIs) were calculated.

**Results:**

A total of 13 phase III RCTs involving 7696 advanced EC patients were included. Pooled analysis showed immunotherapy was associated with favorable outcomes (HR = 0.73; 95% CI, 0.69–0.77; *P* < 0.001). Although both PD-L1-positive and PD-L1-negative patients can benefit from ICIs, there was a robust PD-L1 expression-treatment interaction (*P*_*Interaction*_ = 0.004). Esophageal squamous cell carcinoma (ESCC; HR = 0.73; 95% CI, 0.64–0.84; *P* = 0.001) patients, but not esophageal adenocarcinoma (EAC; HR = 0.92; 95% CI, 0.76–1.13; *P* = 0.44) patients, experienced survival benefits from immunotherapy. Asian patients derived greater benefits (HR = 0.68; 95% CI, 0.62–0.75) than non-Asian patients (HR = 0.81; 95% CI, 0.77–0.91; *P*_*Interaction*_ = 0.03). Notably, ICIs conferred consistent OS benefits across most clinicopathological subgroups categorized based on sex, age, body weight, smoking, ECOG performance status, disease stage, number of metastatic organs, and liver/lung metastasis.

**Conclusion:**

Immunotherapy significantly improves outcomes in advanced esophageal cancer. However, for EAC patients, clinicians must thoughtfully evaluate the treatment effectiveness, safety, and patient preferences to provide personalized management. PD-L1 expression and race tend to be associated with better outcomes rather than acting as a standalone indicator for patient stratification. Other routinely evaluated clinicopathological features showed no significant modulating effect on immunotherapy efficacy.

**Systematic Review Registration:**

Identifier CRD420261348582.

## Introduction

Esophageal cancer (EC) is one of the most serious health issues, ranking seventh in incidence and sixth in cancer-related mortality globally (Siegel et al., 2025). Conventional treatments, including surgery, chemotherapy, and radiotherapy, often have limited effectiveness, particularly in advanced EC (Zhao et al., 2020). In the past decade, the introduction of immune checkpoint inhibitors (ICIs) targeting the programmed cell death protein 1 (PD-1) or its ligand PD-L1 has significantly improved clinical outcomes and transformed the treatment landscape of EC (Sano et al., 2026; Zhang Y. et al., 2024). Currently, immunotherapy, alone or in combination, has become the therapeutic mainstay for advanced EC, encompassing both esophageal squamous cell carcinoma (ESCC) and esophageal adenocarcinoma (EAC) (Zhao et al., 2026).

Even with these advances, only a small fraction of EC patients can benefit from immunotherapy. Additionally, optimal patient selection remains an important clinical challenge (Sano et al., 2026; Ma et al., 2025). Biomarkers with robust predictive power in other types of tumors, such as high tumor mutational burden or microsatellite instability-high status, are rarely detected in EC (Theophilus et al., 2026). Despite extensive investigation, no reliable biomarker for immunotherapy has been established, and currently, personalizing immunotherapy for EC based on biomarkers is still difficult in EC (Ma et al., 2025). Due to the considerable heterogeneities in the pathogenesis of EC, it is essential to thoroughly understand the clinical implications and tackle the debated aspects of immunotherapy before relying solely on it. The clinical and pathological features of EC are widely recognized to significantly influence the success of cancer therapies (Dinerman and Carr, 2026). However, due to the design and limited power of individual randomized clinical trials (RCTs), the treatment differences between patient subgroups characterized by various clinical-pathological features are poorly understood. Here, with the most up-to-date evidence derived from published phase III RCTs, we performed a comprehensive meta-analysis, with overall survival (OS) as the primary endpoint, to explore the potential predictive value of routinely collected information on patients, diseases, and molecular features in EC patients treated with immunotherapy.

## Methods

This meta-analysis was conducted based on the PRISMA guideline (Page et al., 2021) and was registered in PROSPERO (CRD420261348582).

### Search strategy and selection criteria

We searched EMBASE, MEDLINE, Cochrane Library, and Web of Science for studies on advanced EC from inception to April 2026 published in the English language. The search strategy combined relevant Medical Subject Headings terms, EMTREE terms, and free-text keywords related to: immunotherapy (o)esophageal cancer, randomized controlled trial, PD-1, PD-L1, CTLA-4, TIGIT, checkpoint inhibitor. Additionally, we searched ClinicalTrials.gov to identify unpublished or ongoing clinical trials and minimize publication bias. All researchers carried out the initial search independently, carefully reviewed the title and abstract of the manuscript for relevance, and classified the potential studies as excluded or included in the meta-analysis.

Criteria for inclusion and exclusion were set in advance. To be deemed eligible, studies had to meet the following criteria: (1) study design: phase III RCTs irrespective of the blindness; (2) population: patients over 18 years old with histologically confirmed advanced EC; (3) intervention: at least one arm of patients was treated with ICIs regardless of the treatment’s dosage or duration; and (4) outcomes: information regarding overall survival in the entire enrolled group and/or subgroups categorized by different clinicopathological features. The conditions that led to exclusion from this study were: (1) other investigations in this area, including review articles, comments, editorials, pre-clinical papers, retrospective studies, phase I and phase II trials, quality of life research, and cost-effectiveness studies; (2) studies related to pediatric patients or patients with blood-related conditions including leukemia, lymphoma, multiple myeloma, myelodysplastic syndromes, anemia, platelet disorders, coagulation disorders, other hematologic malignancies, and non-malignant hematological diseases; and (3) subjects experiencing autoimmune disorders, and patients taking glucocorticoids or immunosuppressive treatments.

### Data extraction

The following information was independently extracted by all investigators using a predefined form: (1) study details, covering the trial name, masking method, treatment strategies, primary endpoints, and the sample size for the intention-to-treat analysis; (2) the baseline features for the enrolled patients, including sex, age, race, body weight, ECOG performance status, smoking stastus, PD-L1 expression, disease stage, histology subtype, number of organs with metastases, liver metastases status, and lugn metastases status; (3) the outcomes of eligible studies, including the median overall survival time, the hazard ratios (HRs) and their 95% confidence intervals (CIs) for OS overall or subgroups categoried by different clinicopathological features were calculated or extracted directly from the original reports. For trials with more than one publication, only the latest or most thorough report was included.

### Quality assessment

According to the Cochrane risk of bias tool (Higgins et al., 2011), the risk of bias of all the eligible studies was evaluated by six factors including: (1) random sequence generation; (2) allocation concealment; (3) blinding of participants and personnel; (4) blinding of outcome assessment; (5) incomplete outcome data; and (6) selective reporting.

Begg’s funnel plots were visually examined to evaluate publication bias (Begg and Mazumdar, 1994).

In cases where disagreements emerged about study selection, data extraction, or risk of bias assessment, discussions were held among all investigators to address the potential concerns. The discrepancies were resolved once a consensus was reached by all authors.

### Statistical analysis

The primary goals were to investigate the enhancement of overall survival in EC patients with diverse clinicopathological characteristics receiving ICIs. To evaluate the extent of the immunotherapy benefit, we aggregated results using either the fixed-effects inverse-variance-weighted approach or random-effects models, contingent on the degree of heterogeneity. The extent of inconsistency leading to heterogeneity in various trials was evaluated using the *I* ^2^ statistic. Homogeneity was assumed to be invalid for *I* ^2^ values over 50% and *P* values under 0.05. Interaction tests were performed to identify treatment differences and expressed as *P* for interaction. Subgroup analyses were pre-defined to explore possible heterogeneity sources and evaluate the impact of various exclusion criteria on the overall effectiveness of immunotherapy. In this study, the subgroup analysis was performed based on sex (male vs. female), age (<65 years vs. ≥ 65 years), race (Asian vs. non-Asian), body weight (<60 kg vs. ≥ 60 kg), smoking status (current or former smoker vs. never smoked), Eastern Cooperative Oncology Group (ECOG) performance status (ECOG = 0 vs. ECOG = 1), disease stage (locally advanced vs. distantly metastasis), histology subtype (ESCC vs. EAC), PD-L1 expression (PD-L1-positive vs. PD-L1-negative), number of organs with metastases (<2 vs. ≥ 2), liver metastasis (yes vs. no), and lung metastasis (yes vs. no).

All analyses were carried out using Stata (version 17.0). Two-sided *P* < 0.05 was considered statistically significant. All 95% CIs were two-sided.

## Results

### Baseline characteristics

A total of 3503 potentially relevant articles were found in the initial search. Of these, 2177 were excluded due to duplication. After title and abstract screening, 1261 studies were not eligible for inclusion. After a thorough review of the full text, 52 articles were eliminated (Supplementary Figure S1).

Overall, 13 phase III randomized trials with 7696 patients fulfilled the inclusion criteria (Table 1), namely, ASTRUM-007 (Song et al., 2023), ATTRACTION-3 (Okada et al., 2022; Kato et al., 2019), CheckMate 648 (Doki et al., 2022; Kato et al., 2024), ESCORT (Huang et al., 2020), ESCORT-1st (Luo et al., 2021), GEMSTONE-304 (Li et al., 2024), JUPITER-06 (Wang et al., 2022), ORIENT-15 (Lu et al., 2022), RATIONALE-302 (Shen et al., 2022), RATIONALE-306 (Xu et al., 2023), SKYSCRAPER-08 (Hsu et al., 2026), KEYNOTE-181 (Kojima et al., 2020), and KEYNOTE-590 (Metges et al., 2025; Sun et al., 2021). OS was the primary endpoint or co-primary endpoint in all trials. CheckMate 648 was a three-arm RCT, with treatment regimens consisting of nivolumab combined with chemotherapy, nivolumab plus ipilimumab, and chemotherapy alone; the remaining 12 eligible studies were two-arm RCTs. Ten studies mainly focused on advanced ESCC; KEYNOTE-181 (25) and KEYNOTE-590 (Metges et al., 2025; Sun et al., 2021) investigated advanced EC, and ASTRUM-007 (Song et al., 2023) only enrolled PD-L1-positive EC patients. The efficacy of chemo-immunotherapy was examined in eight trials. ICIs were applied as monotherapy in ATTRACTION-3 (Okada et al., 2022; Kato et al., 2019), ESCORT (Huang et al., 2020), RATIONALE-302 (Shen et al., 2022), and KEYNOTE-181 (Kojima et al., 2020). In CheckMate 648 (Doki et al., 2022; Kato et al., 2024) and SKYSCRAPER-08 (Hsu et al., 2026), dual immune checkpoint inhibition was investigated. Of all the enrolled 7696 individuals (age range, 20–90 years), 3506 patients (45.6%) were in the control arm, and 4190 (54.4%) were treated with ICIs. Among them, 687 subjects were treated with pembrolizumab, 582 with tislelizumab, 531 with nivolumab, 526 with camrelizumab, 368 with serplulimab, 358 with sugemalimab, 327 with sintilimab, 325 with nivolumab + ipilimumab, 229 with tiragolumab + atezolizumab, and 257 with toripalimab.

**TABLE 1:** Baseline characteristics of 13 eligible phase III randomized trials.

Study	Masking	Cancer subtype	Treatment	No. Of patients	Median age (range), years	M/F	Median OS (Months)	OS HR (95% CI)
ASTRUM-007 (Song et al., 2023)	Double-blind	PD-L1+ ESCC	Serplulimab + chemotherapy	368	64 (57–68)	317/51	15.3	0.68 (0.53–0.87)
			Placebo + chemotherapy	183	64 (57–68)	153/30	11.8	
ATTRACTION-3 (Okada et al., 2022; Kato et al., 2019)	Open-label	Advanced ESCC	Nivolumab	210	64 (57–69)	179/31	10.9	0.79 (0.64–0.97)
			Chemotherapy	309	67 (57–72)	185/24	8.5	
CheckMate 648 (Doki et al., 2022; Kato et al., 2024)	Open-label	Advanced ESCC	Nivolumab + chemotherapy	321	64 (40–90)	253/68	12.8	0.78 (0.65–0.93)
			Nivolumab + ipilimumab	325	63 (28–81)	269/56	12.7	0.77 (0.65–0.92)
			Chemotherapy	324	64 (26–81)	275/49	10.7	​
ESCORT (Huang et al., 2020)	Open-label	Advanced ESCC	Camrelizumab	228	60 (54–65)	208/20	8.3	0.71 (0.57–0.87)
			Chemotherapy	220	60 (54–65)	192/28	6.2	
ESCORT-1st (Luo et al., 2021)	Double-blind	Advanced ESCC	Camrelizumab + chemotherapy	298	62 (56–66)	260/38	15.3	0.70 (0.56–0.88)
			Placebo + chemotherapy	298	62 (56–67)	263/35	12.0	
GEMSTONE-304 (Li et al., 2024)	Double-blind	Advanced ESCC	Sugemalimab + chemotherapy	358	63 (40–75)	314/44	15.3	0.70 (0.55–0.90)
			Placebo + chemotherapy	182	63 (43–75)	158/24	11.5	
JUPITER-06 (Wang et al., 2022)	Double-blind	Advanced ESCC	Toripalimab + chemotherapy	257	63 (20–75)	217/40	17.0	0.58 (0.43–0.78)
			Placebo + chemotherapy	257	62 (40–74)	220/37	11.0	
ORIENT-15 (Lu et al., 2022)	Double-blind	Advanced ESCC	Sintilimab + chemotherapy	327	63 (57–67)	279/48	16.7	0.63 (0.51–0.78)
			Placebo + chemotherapy	332	63 (56–67)	288/44	12.5	
RATIONALE-302 (Shen et al., 2022)	Open-label	Advanced ESCC	Tislelizumab	256	62 (40–86)	217/39	8.6	0.70 (0.57–0.85)
			Chemotherapy	256	63 (35–81)	215/41	6.3	
RATIONALE-306 (Xu et al., 2023)	Double-blind	Advanced ESCC	Tislelizumab + chemotherapy	326	64 (59–68)	282/44	17.2	0.66 (0.54–0.80)
			Placebo + chemotherapy	323	65 (58–70)	281/42	10.6	
SKYSCRAPER-08 (Hsu et al., 2026)	Double-blind	Advanced ESCC	Tiragolumab + atezolizumab + chemotherapy	229	63 (57–68)	200/29	15.7	0.70 (0.55–0.88)
			Placebo + chemotherapy	232	63 (57–68)	206/26	11.1	
KEYNOTE-181 (Kojima et al., 2020)	Open-label	Advanced EC	Pembrolizumab	314	63 (23–84)	273/41	7.1	0.89 (0.75–1.05)
			Chemotherapy	314	62 (24–84)	271/43	7.1	
KEYNOTE-590 (Metges et al., 2025; Sun et al., 2021)	Open-label	Advanced EC	Pembrolizumab + chemotherapy	373	64 (28–94)	306/67	12.3	0.72 (0.62–0.84)
			Placebo + chemotherapy	376	62 (27–89)	319/57	9.8	

EC, esophageal cancer; ESCC, esophageal squamous cell carcinoma; F, female; M, male; OS, overall survival.

There was a low risk of bias overall among the included RCTs, with the main concern being the absence of blinding. Six studies, including ATTRACTION-3 (Okada et al., 2022; Kato et al., 2019), CheckMate 648 (Doki et al., 2022; Kato et al., 2024), ESCORT (Huang et al., 2020), RATIONALE-302 (Shen et al., 2022), KEYNOTE-181 (Kojima et al., 2020), and KEYNOTE-590 (26, 27), were open-label.

### Overall efficacy of immunotherapy in esophageal cancer

**FIGURE 1:**
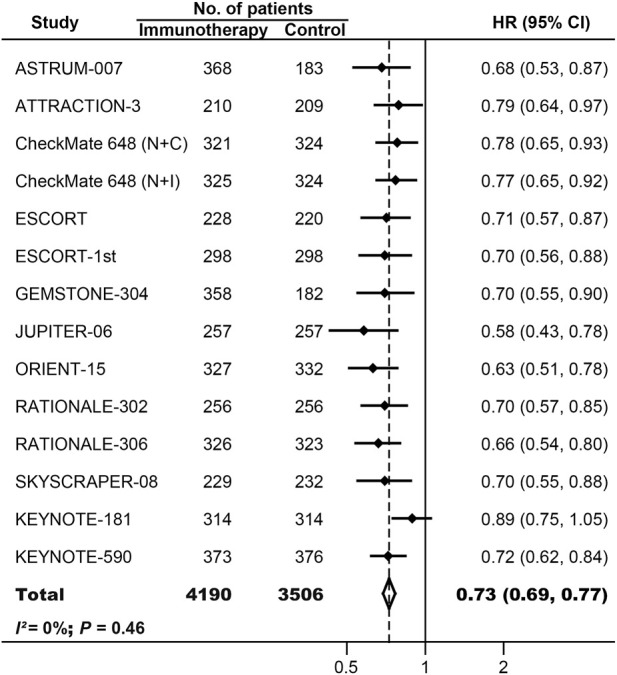
In 7696 patients with esophageal cancer enrolled in 13 phase III randomized controlled trials (RCTs), immunotherapy was associated with favorable overall survival (OS). N + C, Nivolumab + chemotherapy; N + I, Nivolumab + ipilimumab; HR, hazard ratio; CI, confidence interval.

Immunotherapy was associated with favorable OS in twelve studies, but not in KEYNOTE-181 (Kojima et al., 2020). In all 13 phase III RCTs that enrolled 7696 patients, the pooled analysis revealed that ICIs could reduce the risk of death by 27% (HR, 0.73; 95% CI, 0.69–0.77; *P* < 0.001; Figure 1). No significant heterogeneity was observed among these eligible RCTs (*I* ^*2*^ = 0%, *P* = 0.46). Of note, ATTRACTION-3 (Okada et al., 2022; Kato et al., 2019), ESCORT (Huang et al., 2020), RATIONALE-302 (Shen et al., 2022), and KEYNOTE-181 (Kojima et al., 2020) examined the efficacy and safety of mono-immunotherapy as second-line treatment in advanced EC, while the rest of the studies explored the outcomes of a combination of immunotherapy with chemotherapy as first-line treatment. Hence, we further conducted the sensitivity analyses in these subgroups. Both mono-immunotherapy as second-line treatment (HR, 0.78; 95% CI, 0.71–0.86; *P* < 0.001; heterogeneity significant; *I* ^*2*^ = 29%, *P* = 0.24) and chemo-immunotherapy as first-line treatment (HR, 0.70; 95% CI, 0.66–0.75; *P* < 0.001; heterogeneity significant; *I* ^*2*^ = 0%, *P* = 0.79) were associated with longer OS in advanced EC. Additionally, visual examination of Begg’s funnel plot did not reveal any notable asymmetry (Supplementary Figure S2).

### Association between PD-L1 expression and overall survival

**FIGURE 2:**
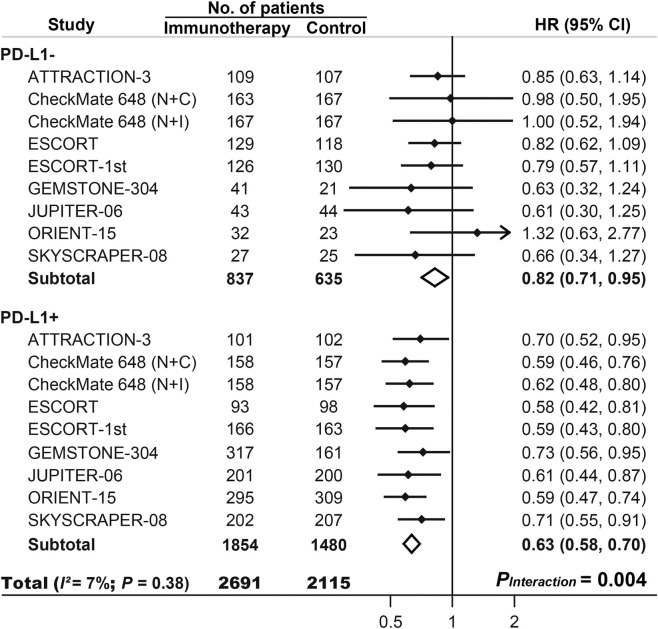
The association between PD-L1 expression and overall survival in patients treated with immunotherapy for esophageal cancer. N + C, Nivolumab + chemotherapy; N + I, Nivolumab + ipilimumab.

The association between PD-L1 expression and OS was reported in eight trials with 4806 subjects. Immunotherapy was associated with longer survival in 3334 PD-L1-positive patients (HR, 0.63; 95% CI, 0.58–0.70; *P* < 0.001) and 1472 PD-L1-negative patients (HR, 0.82; 95% CI, 0.71–0.95; *P* = 0.03). However, these results should be interpreted with caution. As shown in Figure 2, for PD-L1-negative tumors, immunotherapy showed comparable efficacy with controls in every single eligible study. In contrast, individuals with PD-L1-positive diseases could benefit from immunotherapy in all the comparisons. Indeed, there was a robust PD-L1 expression-treatment interaction (*P*_*Interaction*_ = 0.004). Visual inspection of Begg’s funnel plot revealed no obvious publication bias asymmetry (Supplementary Figure S3).

### Impact of histology or race on the immunotherapy outcomes

**FIGURE 3:**
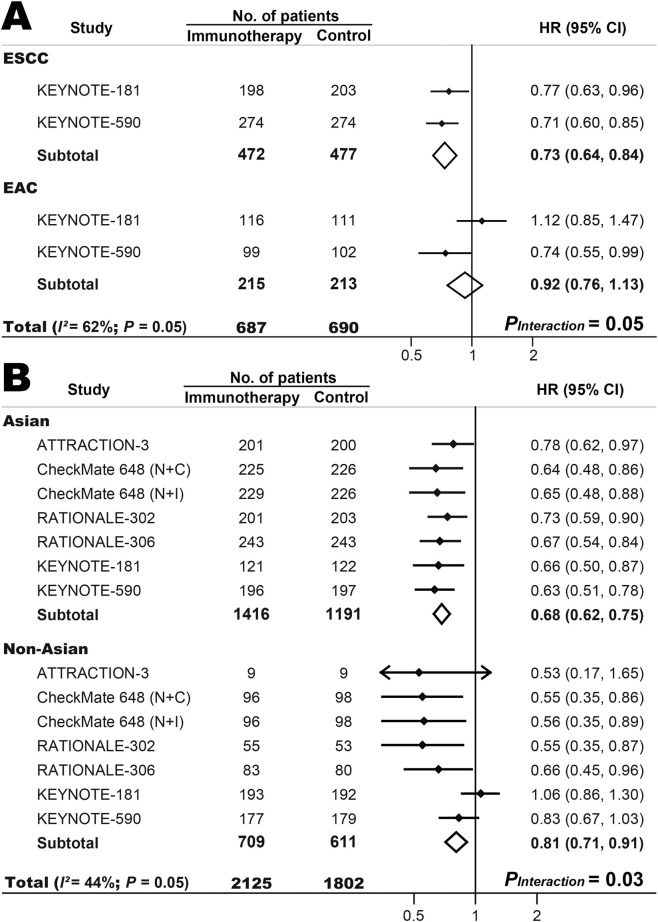
The association between **(A)** histology or **(B)** race and outcomes in patients treated with immunotherapy for esophageal cancer. EAC, esophageal adenocarcinoma; ESCC, esophageal squamous cell carcinoma; N + C, Nivolumab + chemotherapy; N + I, Nivolumab + ipilimumab.

The histological subtypes of EC were reported in two trials with 1377 patients (Figure 3A). Immunotherapy was associated with favorable OS in ESCC (HR, 0.73; 95% CI, 0.64–0.84; *P* = 0.001), but not in EAC (HR, 0.92; 95% CI, 0.76–1.13; *P* = 0.44). Even though it was marginal, the histology-treatment interaction was statistically significant (*P*_*Interaction*_ = 0.05).

It was known that EAC represents over 70% of all EC cases in the United States and Western Europe, while ESCC accounts for 90% of patients in China (Lyu et al., 2025). Here, we also examined whether racial disparities influence the potential benefit of immunotherapy. Racial details were documented in 6 RCTs with 3927 patients (Figure 3B). Although immunotherapy was associated with favorable outcomes in both Asian (HR, 0.68; 95% CI, 0.62–0.75) and Non-Asian (HR, 0.81; 95% CI, 0.77–0.91), the extent of the benefits was significantly different (*P*_*Interaction*_ = 0.03). Additionally, visual examination of Begg’s funnel plot did not reveal any notable asymmetry (Supplementary Figure S4).

### Subgroup analyses by other clinicopathological characteristics

Both male (HR, 0.72; 95% CI, 0.67–0.76) and female (HR, 0.67; 95% CI, 0.57–0.78) patients benefited from immunotherapy, and the benefits were similar between these two subgroups (*P*_*Interaction*_ = 0.41; Supplementary Figure S5). ICIs were associated with longer survival in patients under 65 years old (HR, 0.73; 95% CI, 0.68–0.79) and patients over 65 years old (HR, 0.67; 95% CI, 0.61–0.73). The outcomes were independent of age (*P*_*interaction*_ = 0.15; Supplementary Figure S6). Similar results were observed in patients whose body weight was less than 60 kg (HR, 0.72; 95% CI, 0.59–0.88) and patients over 60 kg (HR, 0.60; 95% CI, 0.47–0.77; *P*_*interaction*_ = 0.26; Supplementary Figure S7), current or former smoker (HR, 0.67; 95% CI, 0.61–0.74) and individuals that never smoked (HR, 0.72; 95% CI, 0.60–0.83; *P*_*interaction*_ = 0.52; Supplementary Figure S8), patients with ECOG = 0 (HR, 0.71; 95% CI, 0.64–0.79) and patients with ECOG = 1 (HR, 0.68; 95% CI, 0.63–0.73; *P*_*interaction*_ = 0.48; Supplementary Figure S9), individuals with locally advanced EC (HR, 0.67; 95% CI, 0.55–0.82) and individuals with distantly metastatic diseases (HR, 0.67; 95% CI, 0.62–0.73; *P*_*interaction*_ = 0.99; Supplementary Figure S10), subjects with less than two metastatic organs (HR, 0.72; 95% CI, 0.62–0.84) and subject with over two metastatic organs (HR, 0.63; 95% CI, 0.54–0.72; *P*_*interaction*_ = 0.20; Supplementary Figure S11), patients withour liver metastasis (HR, 0.67; 95% CI, 0.61–0.75) and patients with liver metastasis (HR, 0.66; 95% CI, 0.55–0.78; *P*_*interaction*_ = 0.81; Supplementary Figure S12), individuals without lung metastasis (HR, 0.58; 95% CI, 0.47–0.72)and individuals with lung metastasis (HR, 0.71; 95% CI, 0.52–0.97; *P*_*interaction*_ = 0.32; Supplementary Figure S13).

## Discussion

Immunotherapy, alone or in combination with other treatments, has gradually replaced conventional management and become the therapeutic mainstay for advanced EC since 2019. Here, based on 13 phase III RCTs with 7696 individuals, for the first time, we explored the impacts of routinely collected characteristics on survival in patients treated with ICIs. Immunotherapy reduces the risk of death by 27% in advanced EC patients. Of note, although our results reveal that both patients with PD-L1-positive tumors and PD-L1-negative tumors can benefit from ICIs, there is a robust PD-L1 expression-treatment interaction. Moreover, histological subtype emerged as a vital biomarker of immunotherapy efficacy, with ESCC patients deriving significant survival benefits while 428 enrolled EAC patients showed no statistically significant improvement in OS. Further examination revealed that Asians benefit more from immunotherapy than Non-Asians. Our study fails to provide sufficient evidence to recommend sex, age, body weight, smoking status, ECOG performance status, disease status, number of metastatic organs, and liver/lung metastasis status as biomarkers for guiding patient selection. These findings may aid in clinical trial design and interpretation, guide treatment decision-making, and promote personalized immunotherapy.

It is widely recognized that tumors lacking PD-L1 expression tend to exhibit diminished or even abrogated antitumor immune responses following ICI administration (Zhang SL. et al., 2024). Here, we demonstrated that PD-L1-negative patients achieved superior clinical outcomes with ICB-based therapy relative to conventional treatment regimens. Several factors may underlie the survival benefits observed in this subpopulation. First, PD-L1 status evaluated using archived tumor specimens collected after disease progression cannot precisely reflect the biomarker profile at the time of initial treatment. Second, qualified tumor tissues available for PD-L1 detection are often limited in clinical practice. Third, heterogeneous PD-L1 expression patterns frequently exist across distinct tumor histological subtypes. Fourth, the PD-L1 detection method and the threshold for PD-L1 positivity or negativity were different among various trials, which may introduce some bias to the conclusion. Fifth, it should be noted that various PD-L1 scoring systems, including combined positive score (CPS) and tumor proportion score (TPS), were applied in different eligible trials. Moreover, the thresholds in different scoring systems were not comparable. This may potentially affect subgroup classification. Moreover, it is well established that chemotherapy, radiotherapy, and even immunotherapy itself can promote PD-L1 upregulation and enhance tumor immunogenicity by optimizing antigen processing machinery as well as strengthening T-cell-mediated cytotoxicity within tumor tissues (Anagnostou et al., 2017; Hegde and Chen, 2020). Mounting clinical and preclinical evidence has confirmed that a broad spectrum of chemotherapeutic agents are capable of triggering immunogenic cell death (ICD) in tumor cells. The immunologically favorable microenvironment remodeled by drug-triggered ICD substantially potentiates the therapeutic efficacy of immunotherapy. Beyond their direct cytotoxic effects on malignant cells, combined chemo-immunotherapy can further activate immune effector cells and reinforce systemic antitumor immune responses. This immunostimulatory effect persists even when primary tumor lesions and tumor-associated antigens remain present throughout the treatment course (Bracci et al., 2014).

It was well-established that EAC and ESCC differed significantly in etiology, pathology, tumor location, therapeutics, and outcomes (Deboever et al., 2024; Lagergren et al., 2017). Indeed, EAC and ESCC exhibit striking and distinct immune microenvironment profiles, which underpin their differential responses to immunotherapy (Lyu et al., 2025). ESCC is characterized by a higher tumor mutational burden, more abundant immune cell infiltration, and a stronger immune-activated tumor microenvironment, which are more conducive to the anti-tumor immune response induced by ICIs (Abnet et al., 2018). It shows significantly higher infiltration levels of immune-effector cells, key cell populations responsible for initiating and mediating anti-tumor immune responses (Zhang et al., 2022). Concurrently, ESCC has a lower proportion of regulatory T cells (Tregs), the immunosuppressive cells that dampen anti-tumor immunity, further reinforcing its pro-immune activation state (Lyu et al., 2025). Additionally, ESCC presents reduced endothelial cell infiltration, linked to impaired tumor angiogenesis and consistent with the enrichment of hypoxia signaling pathways in this subtype; this hypoxia-immune crosstalk forms a unique immune-related molecular landscape in ESCC (Lyu et al., 2025; Zhang et al., 2022). In contrast, EAC is often associated with chronic gastroesophageal reflux and Barrett’s esophagus, with a relatively immunosuppressive TME and fewer tumor neoantigens, leading to poor responsiveness to single-agent or combined immunotherapy (Abnet et al., 2018). It displays dominant Treg infiltration, which suppresses the function of anti-tumor immune cells and creates a tolerogenic immune milieu that facilitates tumor evasion of immune surveillance (Lyu et al., 2025; Zhang et al., 2022). Unlike ESCC, EAC has no significant enrichment of immune-effector cell populations, and its immune landscape is closely coupled with metabolic dysregulation, which further exacerbates immune suppression by altering nutrient availability and immune cell metabolism (Lyu et al., 2025; Siewert and Ott, 2007). Additionally, these two subtypes also differ markedly in immune checkpoint gene expression among 47 examined immune checkpoint genes (Lyu et al., 2025; Zhang et al., 2022). This differential expression of immune regulatory molecules further modulates their respective immune microenvironments and contributes to subtype-specific responses to immunotherapy.

Racial differences in immunotherapy efficacy further reflect the impact of histological subtype and epidemiological background. Since ESCC accounts for over 90% of EC cases in Asia (Li et al., 2021), while EAC is the main subtype in the United States and Western Europe (Deboever et al., 2024), this racial difference is essentially a reflection of the histological subtype distribution. Notably, most key clinical trials exploring new ICIs have been primarily or solely carried out in China. The limited number of EAC patients included in the analysis may have led to an underestimation of immunotherapy efficacy in EAC. Additionally, regional variations in epidemiological profiles, comorbidity loads, treatment sequences, and supportive care standards could hinder the straightforward application of these trial results to other regions. Therefore, although current evidence supports the clinical effectiveness of immunotherapy for ESCC, it is crucial to conduct multinational studies and global validation efforts to ensure its applicability and to develop optimized treatment strategies tailored to various clinical settings around the world. Future studies need to focus on developing personalized immunotherapy regimens for EAC to remodel the immunosuppressive TME.

A striking finding of this study is that immunotherapy conferred consistent survival benefits across most clinicopathological subgroups. This indicates that these factors do not affect the responsiveness to immunotherapy, which has important clinical implications: clinicians do not need to exclude patients from immunotherapy based on these routine clinical indicators. For example, elderly patients (≥65 years), patients with poor physical status (ECOG = 1), and those with multiple organ metastases or liver/lung metastases all achieved significant survival benefits, suggesting that immunotherapy has a broad applicable population in advanced EC.

This meta-analysis has several inherent limitations that need to be acknowledged. First, the included studies are predominantly advanced EC, and the results cannot be directly extrapolated to resectable or early-stage EC patients. The predictive value of clinicopathological characteristics for neoadjuvant/adjuvant immunotherapy in early EC remains to be explored. Second, most trials focused on ESCC, with only a small number of EAC patients included, leading to insufficient statistical power to accurately evaluate its associated predictive factors. Further multinational research and worldwide validation are crucial to determine their applicability and to establish the optimal treatment strategies across diverse clinical settings. Third, the analysis of PD-L1 expression is limited by the lack of unified detection standards, and the impact of different PD-L1 cut-off values on the results cannot be further stratified. Finally, the analysis of adverse events was not performed due to the lack of subgroup-specific safety data, and the balance between efficacy and safety in different subgroups needs to be further investigated.

In summary, this comprehensive meta-analysis confirms that immunotherapy significantly improves overall survival in advanced EC patients. Histological subtype serves as a key predictive biomarker for immunotherapy, as ESCC patients derive significant benefits, while EAC patients show no statistically significant improvement in OS. On the other hand, although immunotherapy confers therapeutic benefits independent of PD-L1 expression levels and race, superior survival gains from immunotherapy were observed in patients harboring PD-L1-positive tumors as well as Asian patients. Notably, immunotherapy confers consistent survival benefits across most routine clinicopathological subgroups, indicating a broadly applicable population in advanced EC. These findings provide important evidence for the personalized clinical application of immunotherapy in EC, guiding clinicians to select optimal treatment regimens based on histological subtype. Future multinational, multi-center clinical trials are essential to verify the generalizability of the results and define optimal treatment strategies for diverse clinical settings, ultimately improving the outcomes of EC patients worldwide.

## Data Availability

The original contributions presented in the study are included in the article/Supplementary Material, further inquiries can be directed to the corresponding author.

## Supplementary material

SUPPLEMENTARY FIGURE S1 Flow-chart diagram of selected randomized controlled trials included in this study.

SUPPLEMENTARY FIGURE S2 In 13 eligible trials with 7696 patients, Begg’s funnel plot for the publication bias test. Each circle represents one trial for the indicated association. Horizontal line, mean effect size.

SUPPLEMENTARY FIGURE S3 Begg’s funnel plot for the publication bias test in PD-L1 subgroup analysis.

SUPPLEMENTARY FIGURE S4 Begg’s funnel plot for the publication bias test in race subgroup analysis.

SUPPLEMENTARY FIGURE S5 The association between sex (male and female) and overall survival in patients treated with immunotherapy **(A)** and Begg’s funnel plot for the publication bias test **(B)**.

SUPPLEMENTARY FIGURE S6 The association between age (<65 years and ≥ 65 years) and overall survival in patients treated with immunotherapy **(A)** and Begg’s funnel plot for the publication bias test **(B)**.

SUPPLEMENTARY FIGURE S7 The association between body weight (<60 kg and ≥ 60 kg) and overall survival in patients treated with immunotherapy for esophageal cancer.

SUPPLEMENTARY FIGURE S8 The association between smoking status (current or former smoker and never smoked) and overall survival in patients treated with immunotherapy for esophageal cancer **(A)** and Begg’s funnel plot for the publication bias test **(B)**.

SUPPLEMENTARY FIGURE S9 The association between ECOG status (ECOG = 0 and ECOG = 1) and overall survival in patients treated with immunotherapy **(A)** and Begg’s funnel plot for the publication bias test **(B)**.

SUPPLEMENTARY FIGURE S10 The association between disease stage (locally advanced and distantly metastatic) and overall survival in patients treated with immunotherapy **(A)** and Begg’s funnel plot for the publication bias test **(B)**.

SUPPLEMENTARY FIGURE S11 The association between the number of organs with metastases (<2 and ≥ 2) and overall survival in patients treated with immunotherapy **(A)** and Begg’s funnel plot for the publication bias test **(B)**.

SUPPLEMENTARY FIGURE S12 The association between liver metastases (yes and no) and overall survival in patients treated with immunotherapy **(A)** and Begg’s funnel plot for the publication bias test **(B)**.

SUPPLEMENTARY FIGURE S13 The association between lung metastases (yes and no) and overall survival in patients treated with immunotherapy for esophageal cancer.
