# Supplementary figures and images for: Associations of PD-L1 expression status, tumor histology and patient ethnic background with immunotherapy outcomes in patients with advanced esophageal cancer
Source: Front Pharmacol. 2026 Jul 17;17:1879335. doi: 10.3389/fphar.2026.1879335 (PMC13423770; doi:10.3389/fphar.2026.1879335)

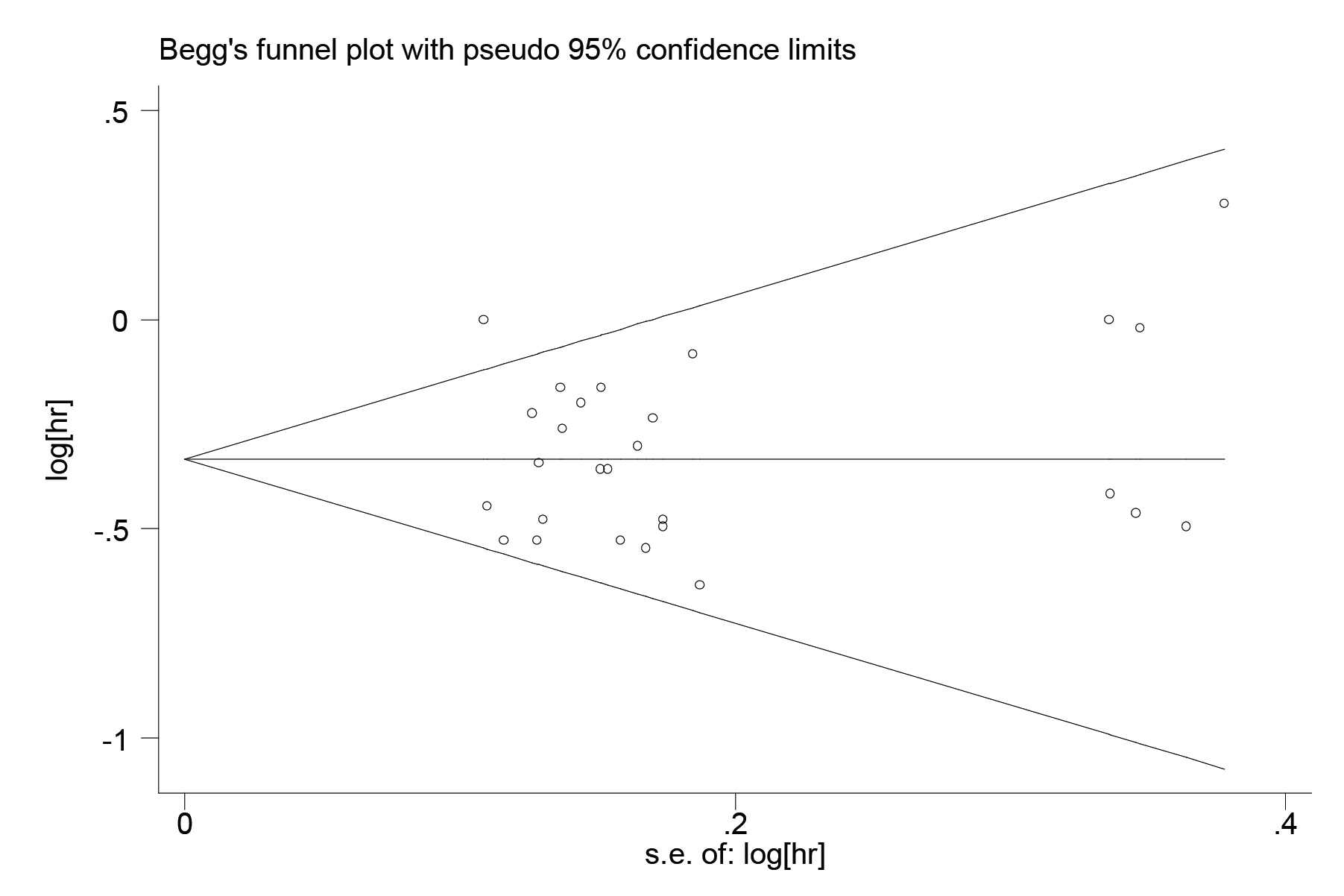

Supplement: Supplementary file 1 [file Image3.jpeg]

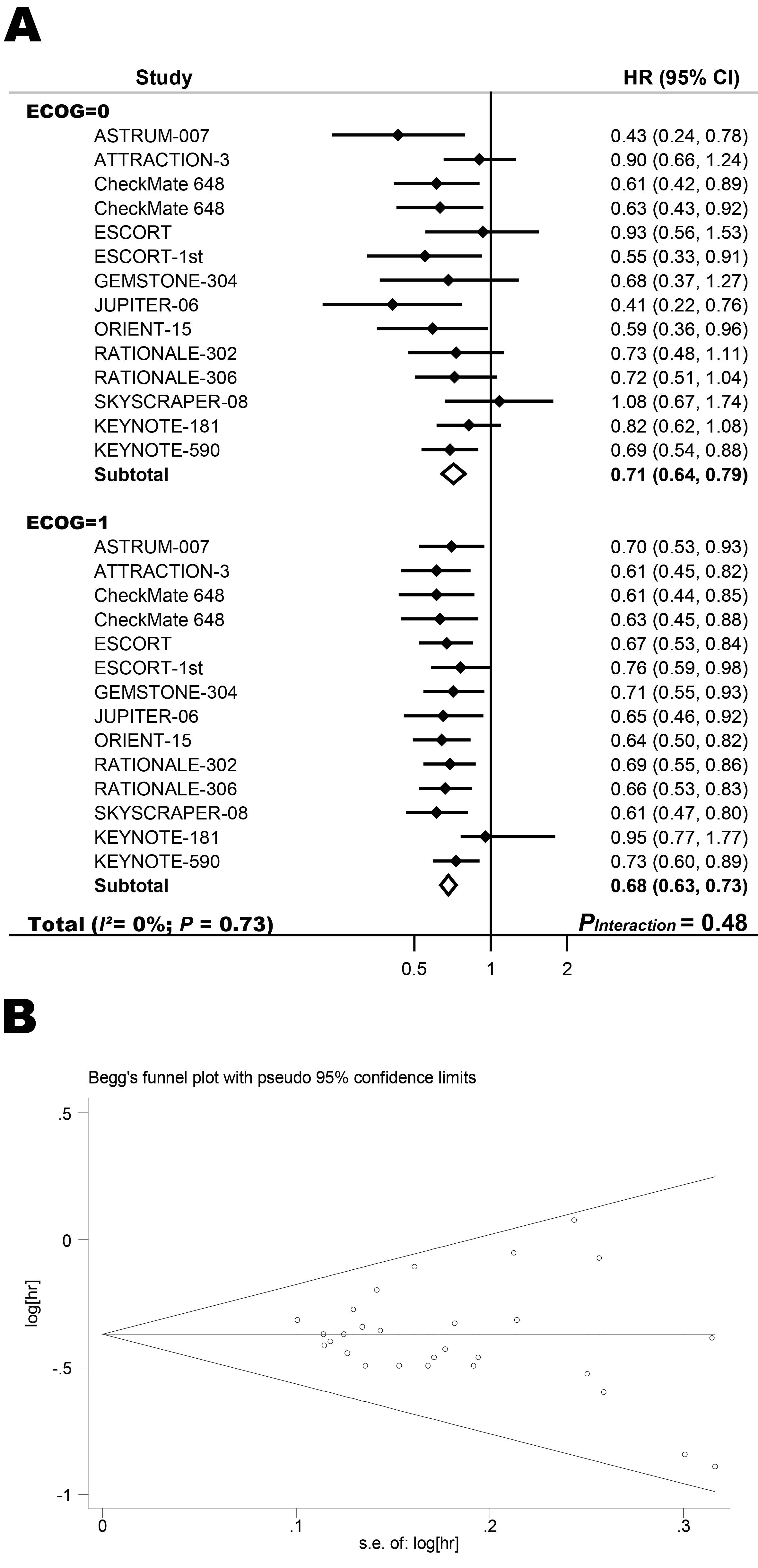

Supplement: Supplementary file 2 [file Image9.jpeg]

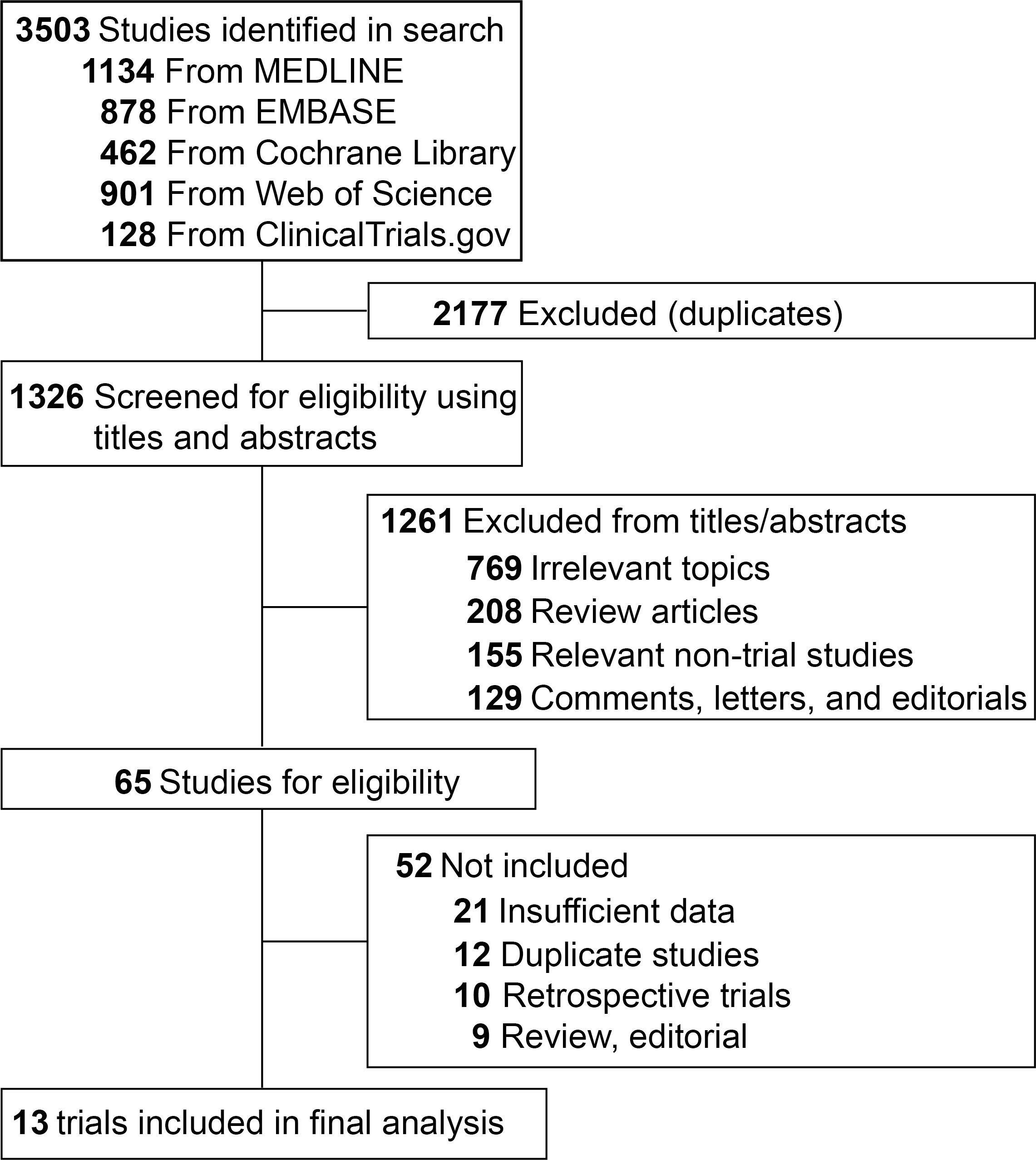

Supplement: Supplementary file 3 [file Image1.jpeg]

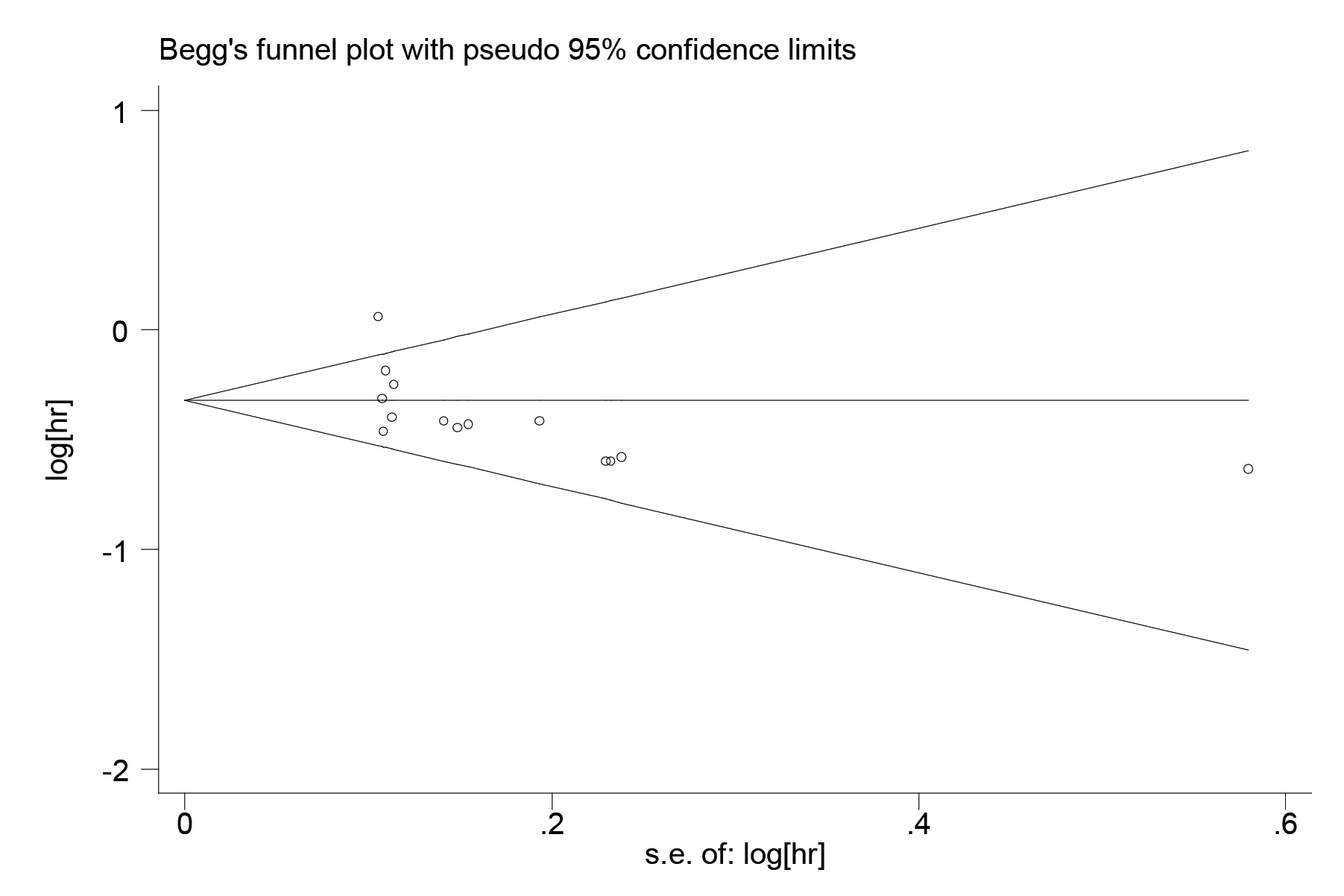

Supplement: Supplementary file 4 [file Image4.jpeg]

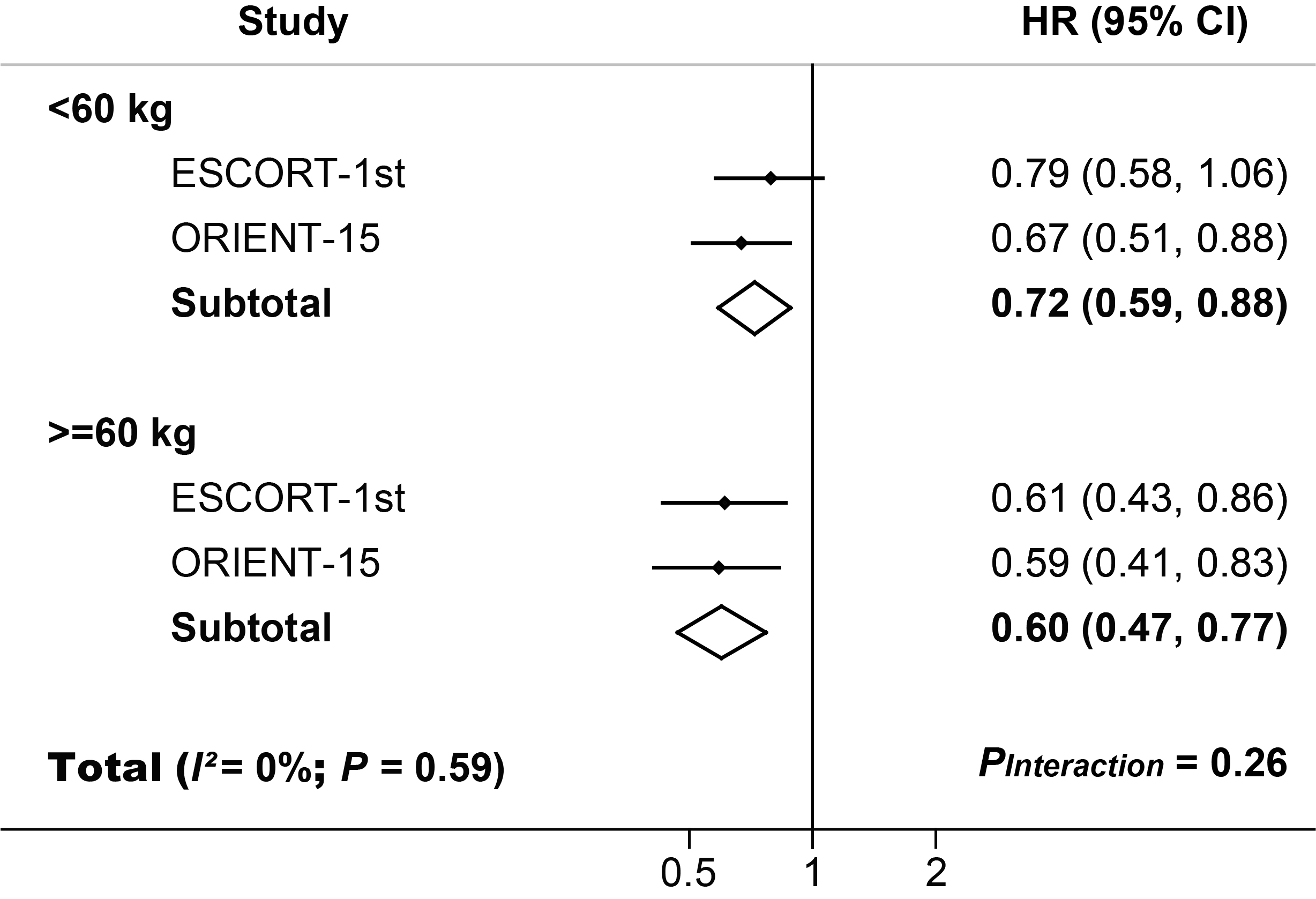

Supplement: Supplementary file 5 [file Image7.jpeg]

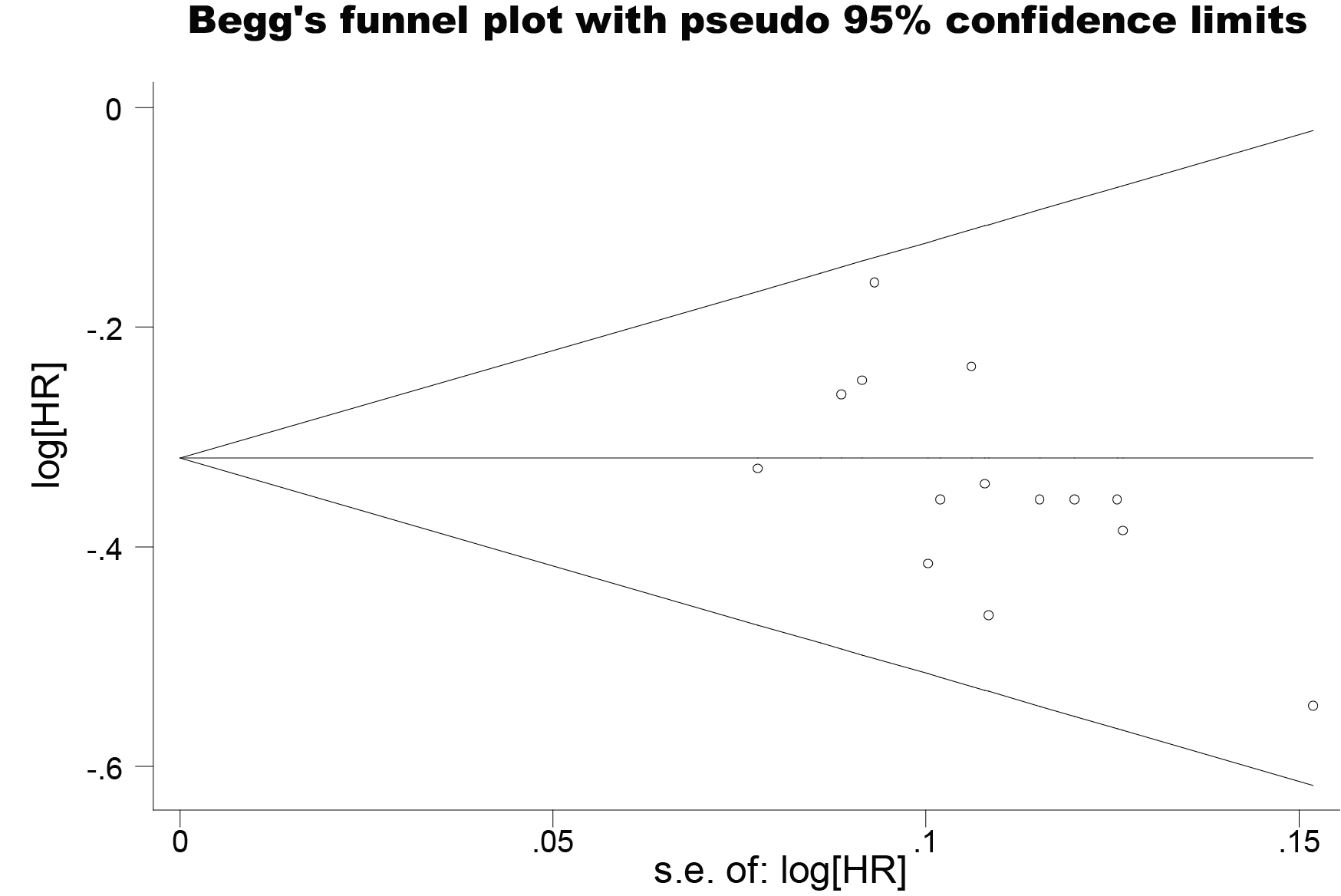

Supplement: Supplementary file 6 [file Image2.jpeg]

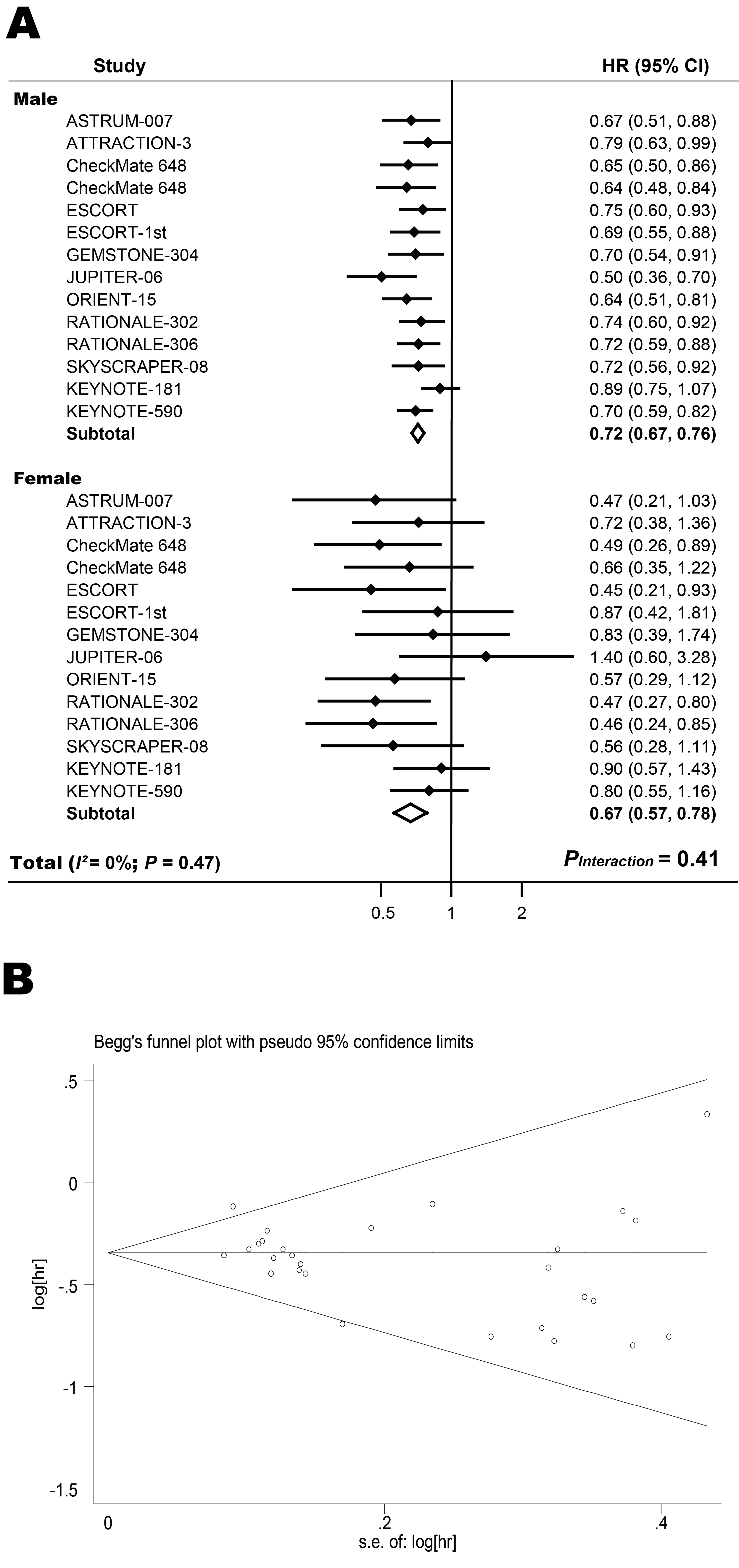

Supplement: Supplementary file 7 [file Image5.jpeg]

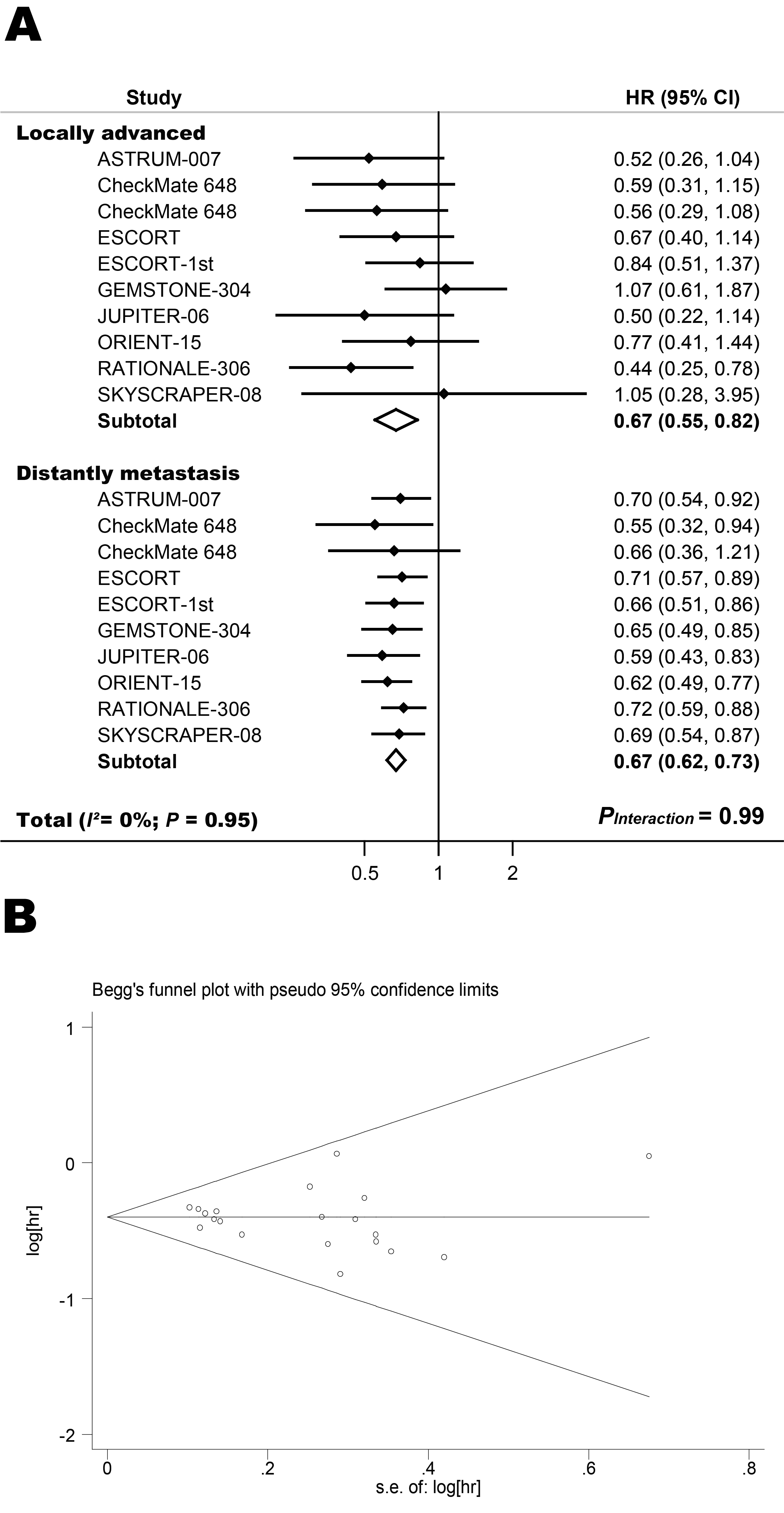

Supplement: Supplementary file 8 [file Image10.jpeg]

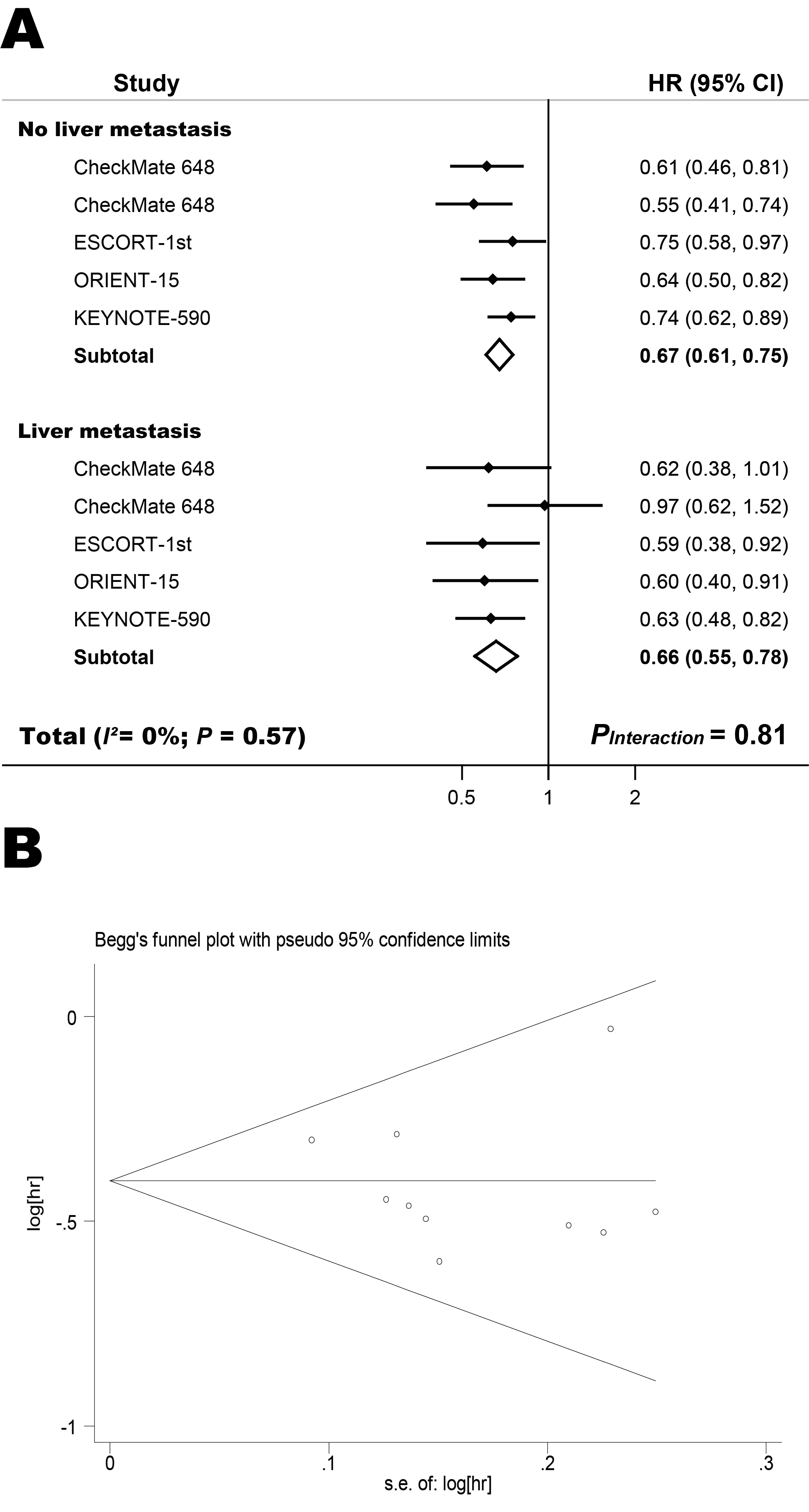

Supplement: Supplementary file 9 [file Image12.jpeg]

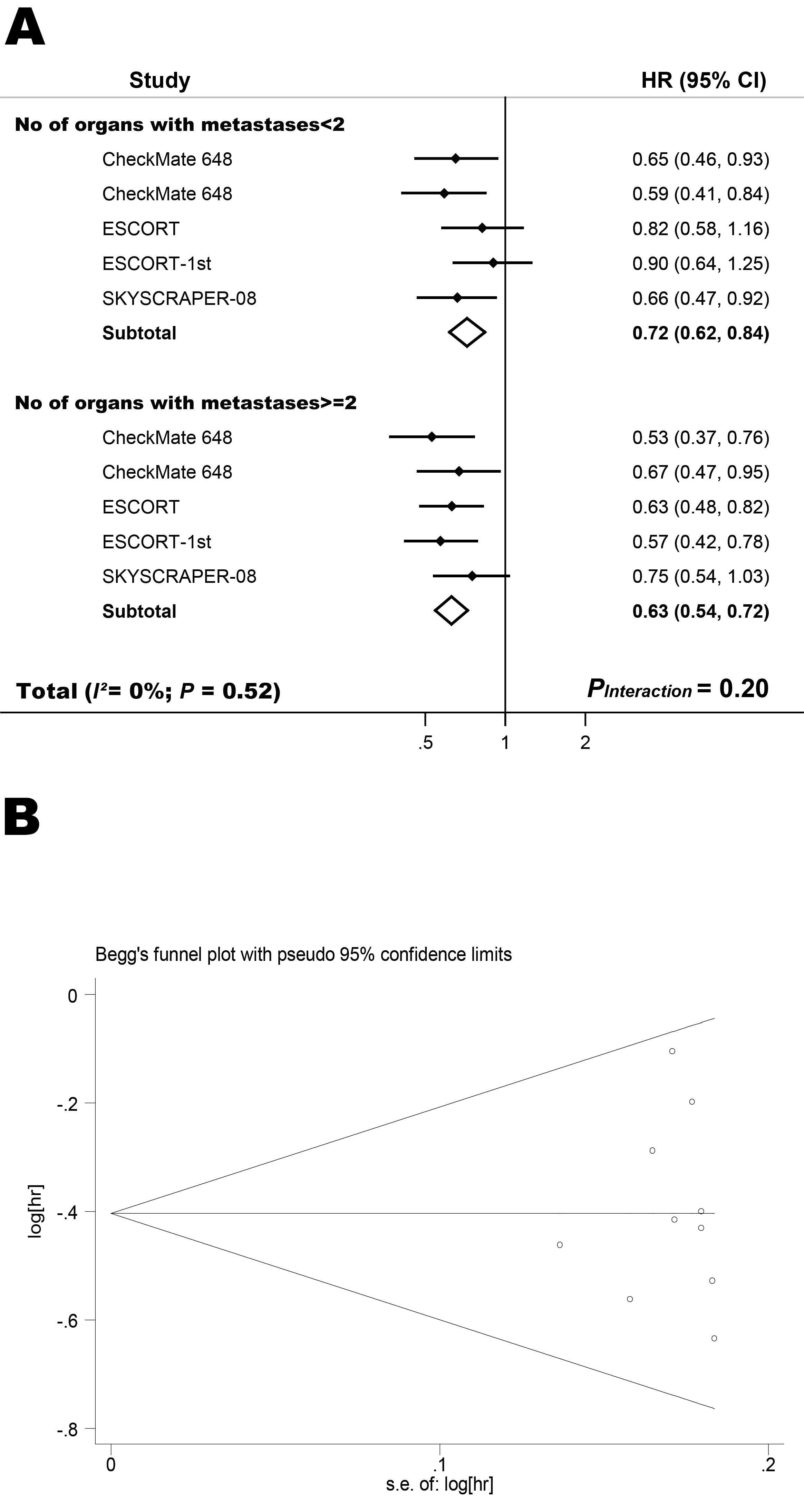

Supplement: Supplementary file 10 [file Image11.jpeg]

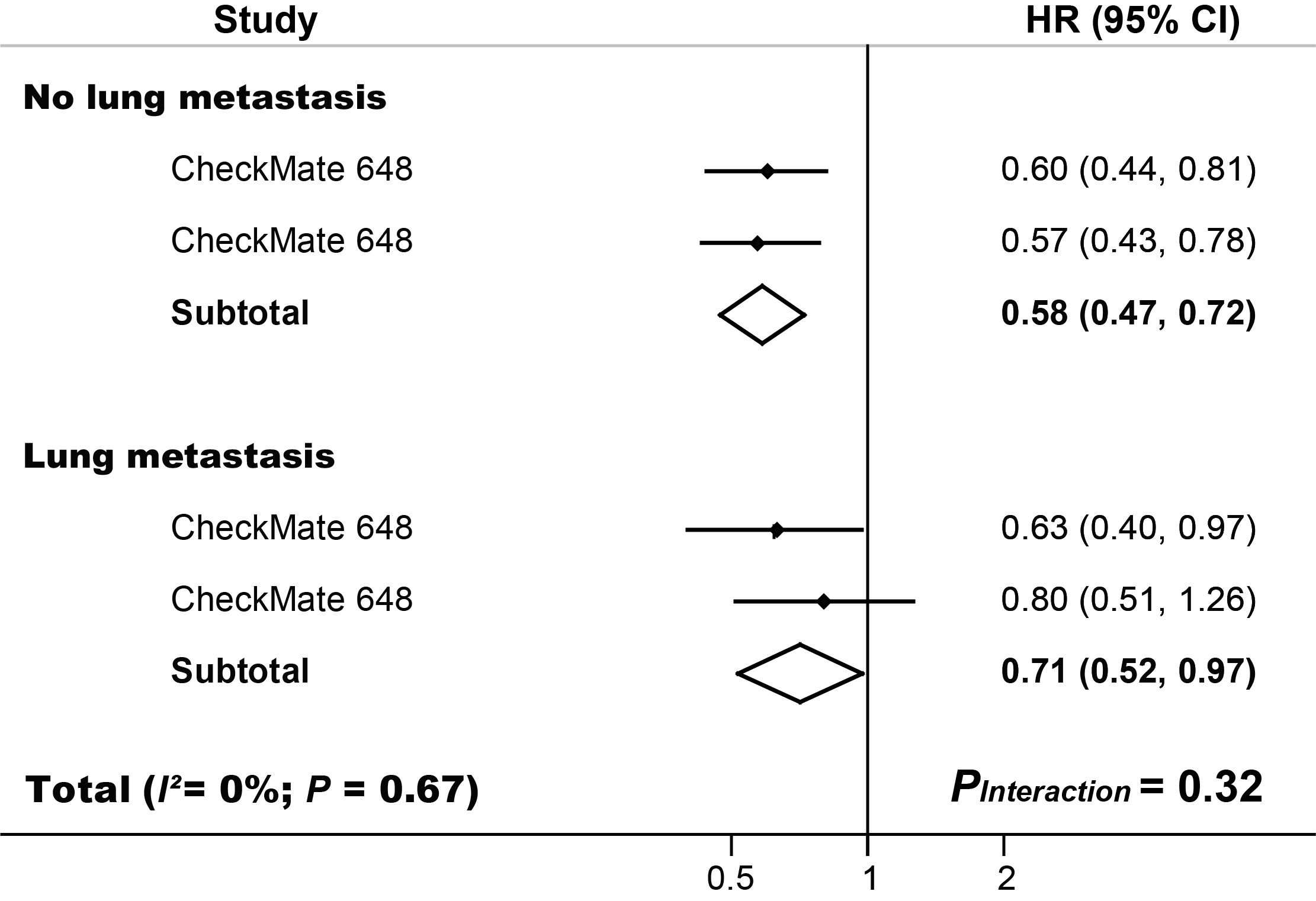

Supplement: Supplementary file 11 [file Image13.jpeg]

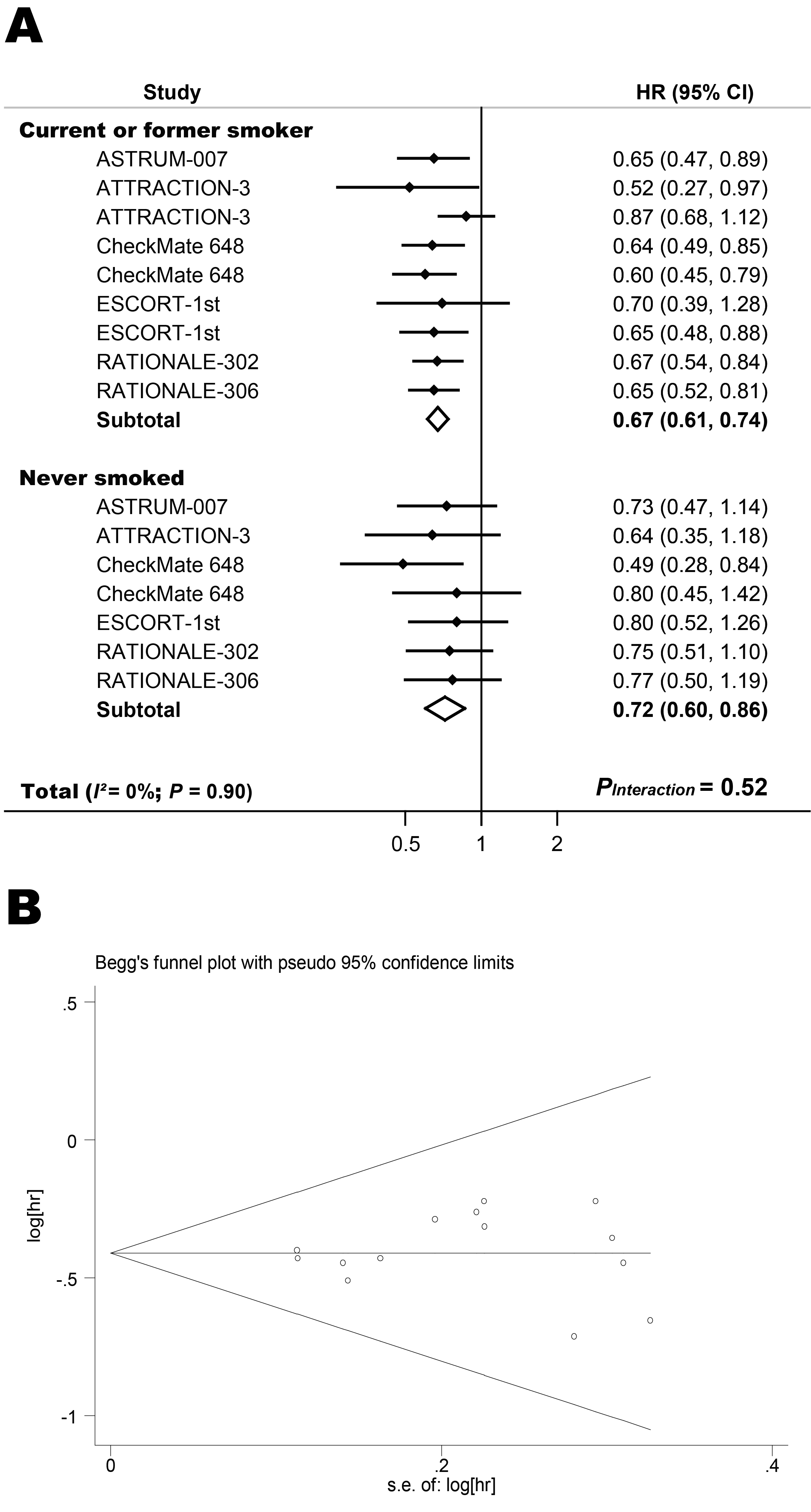

Supplement: Supplementary file 12 [file Image8.jpeg]

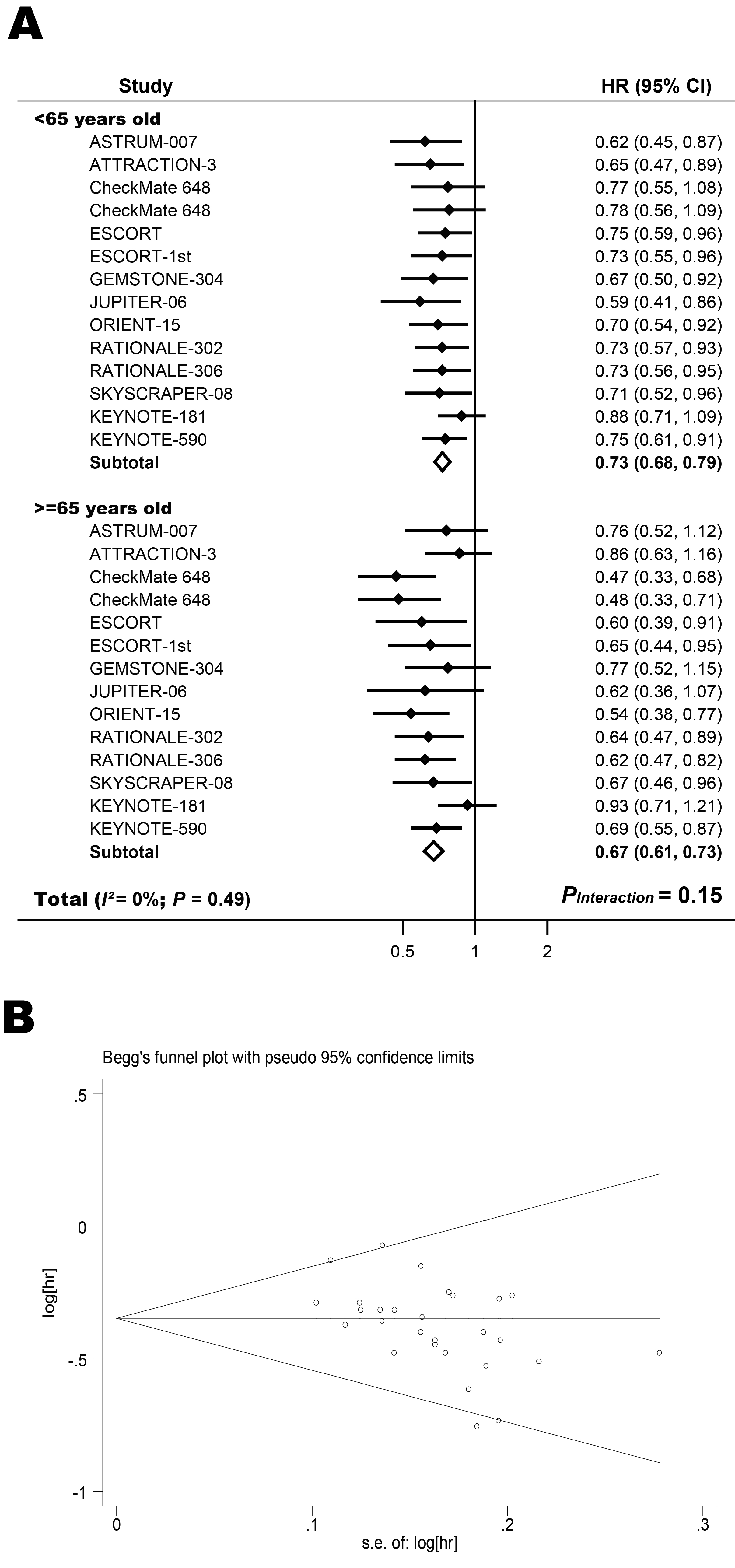

Supplement: Supplementary file 13 [file Image6.jpeg]
